# Visual rating of brain atrophy in structural MRI: Is its time over?

**DOI:** 10.1007/s00330-025-11424-4

**Published:** 2025-02-07

**Authors:** Ralph Buchert

**Affiliations:** Department of Diagnostic and Interventional Radiology and Nuclear Medicine, University Medical Center Hamburg-Eppendorf, Hamburg, Germany

Cognitive impairment can have many different causes, and it can be difficult to distinguish between them clinically, particularly in the case of mild symptoms in early disease stages. According to European public health guidelines, patients with cognitive impairment “should have access to accurate diagnosis at a time in the disease process when it can be of most benefit to them” [1]. With the advent of disease-modifying therapies for primary neurodegenerative diseases [2], this includes reliable determination of the underlying pathology to properly select patients for disease-modifying therapies that target specific pathological processes.

Structural MRI of the brain is often performed as part of the standard diagnostic work-up in patients with clinically uncertain cognitive impairment (CUCI). Traditionally, the primary aim of structural MRI in CUCI has been to detect or exclude secondary causes such as space-occupying lesions, stroke and hydrocephalus (exclusionary approach). However, numerous studies have shown that structural MRI can also be used to detect and differentiate primary neurodegenerative diseases. This is based on disease-specific atrophy patterns that occur as macroscopic downstream sequelae of the primary proteinopatic changes in the brain. This is true not only for classical dementing proteinopathies such as Alzheimer’s disease (AD) and the various subtypes of frontotemporal lobar degeneration (FTLD), but also for proteinopathies that have only recently received widespread attention, such as limbic-predominant age-related TDP-43 encephalopathy (LATE) [3]. This is recognised by current clinical guidelines for the diagnosis of cognitive impairment, which recommend structural MRI to assess not only vascular lesions, but also regional atrophy to directly support the (differential) diagnosis of primary neurodegenerative diseases [4]. This integrative approach does not directly require additional diagnostic procedures and can therefore be very cost-effective. Thus, despite the increasing availability of more specific blood-, CSF-, and PET-based biomarkers, integrative structural MRI approaches will most likely continue to play an important role in the diagnosis of CUCI [5]. In particular, the assessment of regional atrophy on structural MRI could be used to direct patients to the most efficient biomarker pathway to achieve a sufficiently reliable aetiological diagnosis.

Regional atrophy can be assessed on structural MRI either semi-quantitatively using visual rating scales or quantitatively using planimetric or volumetric measures. Visual rating scales may be limited in clinical practice by considerable inter-rater variability and low sensitivity to detect subtle atrophy in the early stages of disease or changes in brain volume on longitudinal imaging. In addition, visual rating scales usually focus on a single brain region. For example, the widely used medial temporal lobe atrophy (MTA) scale proposed by Scheltens and colleagues is restricted to the medial temporal lobe [6]. It is difficult to visually detect mild expression of spatially distributed atrophy patterns involving several distant brain regions. These limitations prompted the development of tools for fully automated volumetric assessment of a whole set of predefined regions of interest in structural MRI [7]. In the second step, the resulting volumetric profiles can be automatically categorised into predefined disease categories using machine learning-trained software tools, either using conventional techniques such as support vector machines or deep learning. Many studies have reported that automated methods are superior to visual rating scales. Does this mean that the time for visual rating is over?

At present, the answer is: not yet! The reason is that many studies on automated volumetry and/or automated categorisation of structural brain MRI have been performed in highly selected patient cohorts that are not representative of clinical practice. Thus, there is a lack of data on the impact of machine learning-based tools on MRI-based detection and differentiation of neurodegenerative diseases in widespread everyday clinical routine, not only in terms of accuracy, but also in terms of usefulness, clinically meaningful outcomes and cost [8]. Indeed, the validation of automated tools for widespread use in clinical practice presents major challenges, not least because MRI-based brain volumetry is often sensitive to the MR scanner platform and to details of the acquisition sequence [9]. In line with the relative lack of clinical validation, the recently updated German S3 Practice Guideline on Dementia does not recommend the use of automated analysis procedures for structural MRI in the context of CUCI without visual assessment by an experienced reader [4]. The guideline explicitly recommends the use of visual rating scales [4], as do many other national and international guidelines and task forces. Furthermore, automated analysis tools often require 3D images with high (more or less isotropic) resolution, which are not yet standard in clinical practice at all sites.

Against this background, the visual 3-level (no, mild, and severe) “Amygdala Atrophy Scale” (ASS) proposed by Pizzini and colleagues in this issue of *European Radiology* to characterise amygdalar atrophy on T1-weighted brain MRI is expected to have a strong impact on current clinical practice [10]. The ASS is determined from two image planes, one axial and one coronal. The authors demonstrate near-perfect within-rater and substantial between-rater stability of the ASS and a strong association with automated Freesurfer estimates of amygladar volume. In addition, the ASS was associated with cognitive status and, at trend level, with LATE neuropathological changes in the amygdala detected at autopsy on average 3 years after MRI. Finally, the authors show that the ASS is not redundant with the MTA scale, which is based on the width of the choroidal fissure and temporal horn of the lateral ventricle and the height of the hippocampus [6]. In particular, the highest MTA score (most severe medial temporal atrophy) did not predict the most severe amygdala atrophy according to the ASS. Thus, the ASS has the potential to improve the visual differentiation between diseases in which temporomesial atrophy is more pronounced in the hippocampus, such as AD, and diseases with more pronounced amygdala involvement, such as semantic variant FTLD or LATE. The latter is increasingly recognised as a relevant aetiology of CUCI in older people.

Future work will include testing the ASS against clinical data to demonstrate its clinical validity, for example in distinguishing LATE from AD. In addition, the current 3-level version of the rating scale could be extended to 4 or 5 levels to better account for age-related volume loss. This could improve the interpretation of intermediate scores and the definition of cut-offs on the ASS for specific clinical tasks.

However, the arguments against the exclusive use of automated procedures for the analysis and interpretation of structural MRI in clinical practice may be resolved as a matter of time rather than feasibility. The exclusive use of fully automated methods may then become the most cost-effective way to reliably assess atrophy on structural MRI to provide diagnostic and predictive information. This is particularly true for deep learning-based methods, which outperform traditional machine learning methods in many medical imaging tasks.
